# metaLoc: protein localisation prediction workflow

**DOI:** 10.1093/bioadv/vbag169

**Published:** 2026-06-19

**Authors:** Conor J R Scott, Silvia Caccia

**Affiliations:** Department of Biosciences, University of Milan, Milan, 20133, Italy; Department of Biosciences, University of Milan, Milan, 20133, Italy

## Abstract

**Summary:**

metaLoc combines existing tools for signal peptide, localisation, and transmembrane helices prediction from protein sequences into a workflow for rapid evaluation of protein datasets. By accepting both protein and nucleotide sequences, the workflow is especially suitable for *in silico* screening of the growing volumes of sequencing data. With a single command, metaLoc provides a simple, accessible, and user-friendly tool for the bioinformatic investigation of proteomic or metagenomic datasets.

**Availability and implementation:**

metaLoc is freely available on the GitHub platform (https://github.com/scottc-bio/metaLoc). The metaLoc workflow is implemented in Nextflow with a modular design utilizing isolated Conda environments for reproducibility. An archived version of this release is permanently available at Zenodo (https://doi.org/10.5281/zenodo.18936772).

## 1 Background

Investigating proteins allows the fundamental principles of biological life to be understood, the effectors of disease to be identified, the complexity of microbial community interactions to be elucidated, and revolutionary biotechnological processes to be developed, alongside many other potential applications. DNA sequence data and proteomic data have rapidly accumulated in public databases (Muir *et al.* 2016, Dai *et al.* 2021), signalling the advancing technology and decreasing costs associated with these experiments. However, the growing volumes of such data have resulted in a deepening complexity of the analysis required to evaluate and understand the systems studied. Proteins of interest may only represent a tiny fraction of the proteome captured in a proteomics experiment, or by a handful of encoding genes in the multiple megabases of sequencing data obtained in a metagenomics experiment. Often bioinformatic tools are relied upon to provide researchers with clues directing to proteins with characteristics similar to those of interest, or to reduce the complexity of datasets by eliminating proteins with unsuitable characteristics for the research goal.

Multiple publicly available tools exist that can predict secretion signals, localisation, or transmembrane helices based on amino acid sequences (Käll *et al.* 2007, Hallgren *et al.* 2022, Teufel *et al.* 2022, Moreno *et al.* 2024, Ødum *et al.* 2024). These tools are invaluable for researchers exploring large datasets, and can often be used via web servers or local command-line execution. The unique methods employed by each tool suggest that combining their predictions is important when forming an assessment of a protein’s characteristics. In reality this is difficult due to the standalone nature of the tools and their diversely structured outputs. Additionally, the web servers are limited in their capacity to handle the large datasets which have become common in biological investigations. Therefore, local installation and setup is required for each individual tool, which can be intimidating and thus inaccessible for researchers with limited bioinformatic experience.

The prediction of *in silico* secretomes, defined as the proteins secreted by a cell or organism, is an example of where these challenges have attempted to be addressed. For example, workflows for combining multiple tools have been suggested (Scott *et al.* 2023) and even implemented in R previously (Gogleva *et al.* 2018). Building upon the workflow suggested previously (Scott *et al.* 2023), metaLoc combines up to date publicly available tools for signal peptide, localisation, and transmembrane helices prediction from both eukaryotic and prokaryotic sequences. With only a single line of code, the metaLoc workflow performs all the necessary installation, isolated environment setup for reproducibility, processing, and output modification and merging to produce a final results table that provides users with the predicted characteristics of their protein datasets in a user-friendly format ready for downstream analysis. Perhaps the most important advancement implemented in the metaLoc workflow is the acceptance of metagenomic nucleotide sequences, from which protein sequences can be predicted, as metagenomic approaches have emerged as a valuable reservoir for protein discovery (Hou *et al.* 2022). The tools for nucleotide sequence classification and protein sequence prediction are activated through modification of the arguments in the single command used to operate metaLoc and all installations occur automatically. Through Nextflow implementation, a rapid and automated workflow seamlessly combines and merges the outputs of all tools to provide users with a comprehensive assessment of per-protein predictions ready for downstream filtering and analysis.

## 2 metaLOC

### 2.1 Design

**Figure 1:**
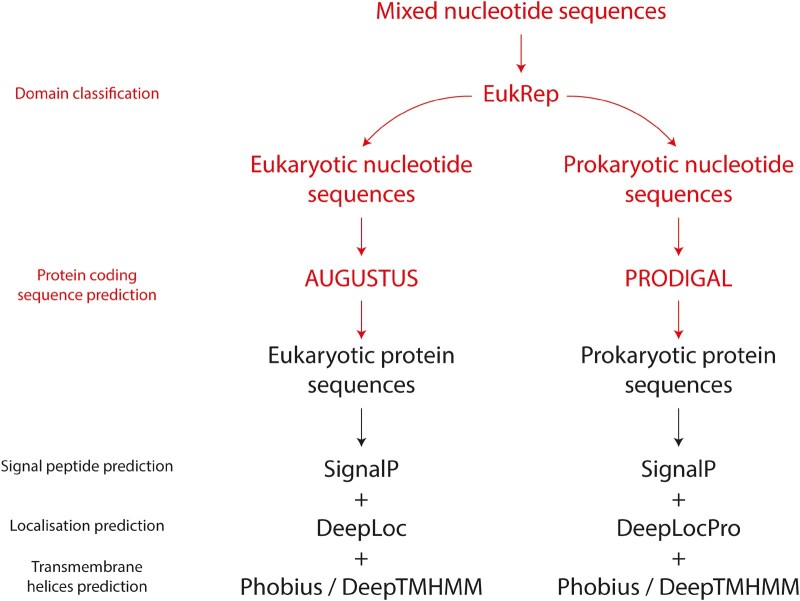
metaLoc workflow design. Metagenomic sequences of mixed origin are classified to eukaryotic and prokaryotic domains with EukRep. Protein amino acid sequences are predicted from eukaryotic nucleotide sequences with AUGUSTUS and from prokaryotic nucleotide sequences with Prodigal. Signal peptide prediction is performed with SignalP. Localization prediction is performed by DeepLoc for eukaryotic protein sequences and by DeepLocPro for prokaryotic protein sequences. Transmembrane helices prediction is performed with either Phobius or DeepTMHMM as specified by the user. Black = core workflow, Red = metagenomic mode.

The metaLoc workflow can be seen in Fig. 1. The core workflow performs signal peptide, localisation, and transmembrane helices prediction with protein sequences accepted as inputs. SignalP 6.0 (Teufel *et al.* 2022) is utilized for signal peptide prediction in ‘euk’ or ‘other’ mode for eukaryotic or prokayotic sequences, respectively. Localisation prediction is performed using DeepLoc 2.1 (Ødum *et al.* 2024) for eukaryotic sequences or DeepLocPro 1.0 (Moreno *et al.* 2024) for prokaryotic sequences. Transmembrane helices prediction can be performed for sequences of both life domains with either Phobius 1.01 (Käll *et al.* 2007) or DeepTMHMM 1.0 (Hallgren *et al.* 2022). DeepTMHMM has been show to outperform Phobius and has the added ability of beta-barrel prediction (Hallgren *et al.* 2022). However, here DeepTMHMM relies on sequence upload to a remote web-based server for prediction and therefore may be unsuitable for large or sensitive datasets. Therefore, users may prefer to enable DeepTMHMM for smaller sets of proteins, or to check the TM prediction of Phobius for subsets of proteins of interest. When the optional metagenomic mode is activated AUGUSTUS 3.5.0 (Hoff and Stanke 2019) can be used to predict protein coding regions from eukaryotic nucleotide sequences, Prodigal 2.6.3 (Hyatt *et al.* 2010) can be used to predict protein coding regions from prokaryotic nucleotide sequences, or EukRep 0.6.7 (West *et al.* 2018) can be used to classify nucleotide sequences of mixed or unknown origin to eukaryotic or prokaryotic domains prior to simultaneously running both branches of the workflow.

### 2.2 Implementation

The metaLoc workflow was implemented in Nextflow 25.10.2 and tested in version 25.10.4. The workflow relies on isolated conda environments for reproducibility and therefore requires Miniconda, conda, or Mamba to be installed. This enables portability across Linux servers, HPC environments, macOS, and Windows Subsystem for Linux (WSL). metaLoc is freely available at: https://github.com/scottc-bio/metaLoc, alongside full instructions on installation and usage. The Nextflow structure allows conda environment setup for all tools of the workflow to occur automatically using the provided files in the ‘envs/’ directory of the metaLoc repository. These environments are initialized once upon first use and are cached by Nextflow for all subsequent runs of the workflow.

### 2.3 Usage

The full list of parameters can be seen in Table 1. The ‘–fasta’ parameter is used to direct the workflow to the user’s input sequences in fasta format. The ‘–organism’ parameter can be set to ‘euk’ for eukaryotic or ‘other’ for prokaryotic amino acid sequences and signals to only run the core workflow of signal peptide, localisation, and transmembrane helices prediction. By default the ‘–meta’ parameter is ‘false’ but can be set to ‘euk’ for AUGUSTUS coding sequence prediction from eukaryotic nucleotide sequences, to ‘other’ for Prodigal coding sequence prediction from prokaryotic nucleotide sequences, or to ‘mixed’ for EukRep domain classification of nucleotide sequences of mixed or unknown origin followed by both branches of the workflow. Ab initio gene prediction with AUGUSTUS is highly organism-specific (Hoff and Stanke 2019), and therefore an appropriate gene model must be selected using the ‘–augustus_model’ parameter. All available models can be viewed online (https://github.com/scottc-bio/metaLoc/blob/main/assets/augustus_model_list.txt) or from the root of the workflow repository with: ‘cat assets/augustus_model_list.txt’.

**Table 1:** metaLoc parameters.

Parameter	Required	Default	Description
—fasta	Yes	—	Input FASTA file containing protein or contig sequences
—organism	Yes*	—	Organism type ('euk’ for eukaryotic sequences or 'other’ for prokaryotic sequences). Required when '—meta’ is false
—meta	No	false	Enables metagenomic mode ('euk’, 'other’, or 'mixed’)
—augustus_model	Conditional	—	Augustus species model. Required when '—meta’ is 'euk’ or 'mixed’
—min_contig_len	Conditional	—	Minimum contig length for EukRep filtering. Required when '—meta’ is 'mixed’
—deeptmhmm	No	false	Use DeepTMHMM instead of Phobius for TM prediction (set to 'true’)
—signalptar	No	assets/signalp-6.0i.fast.tar.gz	Path to SignalP archive
—deeploctar	No	assets/deeploc-2.1.All.tar.gz	Path to DeepLoc archive.
—phobiustar	No	assets/phobius101_linux.tgz	Path to Phobius archive.

All potential parameters used to run metaLoc.

EukRep classification is unreliable on short sequences and therefore sequence lengths of at least 3000 bp are recommended (West *et al.* 2018). Indeed, during testing it was observed that eukaryotic contigs below this length were misclassified as prokaryotic. The ‘–min_contig_len’ parameter allows the user to set the minimal length of nucleotide sequences inputted to EukRep and is set to 3000 by default. To switch the TM predictor to DeepTMHMM, the optional parameter ‘–deeptmhmm’ can be set to ‘true’. The final parameters ‘–signalptar’, ‘–deeploctar’, and ‘–phobiustar’ are used to direct the workflow to the archive files required for these tools. These tools are under academic license and cannot be distributed here, but can be easily obtained by users as explained in the GitHub repository: https://github.com/scottc-bio/metaLoc.

After cloning the repository the test profile can be run. The test run utilizes a fasta file containing the nucleotide sequences of the first 10 Kbp of the first contigs of the *Aspergillus nidulans* var. *acristatus* (GCA_047715555.1) and *Escherichia coli* BPE091041 genome assemblies (GCA_977857295.1). The test profile will run the full workflow in ‘mixed’ meta mode to initially classify the sequences before both branches of the workflow are run simultaneously to predict a single eukaryotic and nine prokarytic protein coding sequences for signal peptide, localisation and TM helices prediction, with Phobius as the TM predictor.

Workflow development and testing was performed on a shared Linux server with Ubuntu 18.04.6 LTS with dual Intel Xeon Gold 6130 processors. In total, 64 CPUs and 248 GB RAM were available. Execution of Nextflow v25.10.4 workflows utilised process parallelisation with available CPU threads. Processes of the workflow were allocated 4 CPUs and 8 GB RAM by default. On this system the test profile took 6 minutes and 41 seconds, including the automatic environment setup. Running the profile for a second time negates any need for environment set up, as these are cached by nextflow, and took 1 minute 46 seconds. Switching to the DeepTMHMM predictor took 7 minutes and 8 seconds with environment setup, and 2 minutes and 24 seconds when utilising cached environments.

To demonstrate the scalability of this workflow to metagenomic applications, the workflow was applied in ‘mixed’ meta mode to a coassembly of mixed microbial contigs with a minimum contig length of 3000 bp selected. In total, 96 091 contig sequences were submitted to the workflow. EukRep classified 7029 as eukaryotic, from which AUGUSTUS predicted 13 953 protein coding sequences. EukRep classified 89 062 as prokaryotic, from which Prodigal predicted 1 380 482 protein coding sequences. Therefore, 1 394 435 protein coding sequences were processed in total. This took 10 days 13 hours 23 minutes and 20 seconds to complete, providing predicted protein characteristics for over 1 million protein sequences encoded within the metagenomic sequences of a mixed microbial community from a single command.

### 2.4 Interpreting results

Each set of input protein sequences will produce two output directories within the ‘results/’ directory, both named according to the input file name. The first contains the full outputs of all tools utilised during the workflow in subdirectories for each tool, the second contains two tab-separated files: “final_merged.tsv” containing the results columns from all tools with one line per input protein sequence, and “final_concise.tsv” containing only the key result columns from each tool. All potential output columns of the final concise results tables can be found in Table 2. These merged results files allow easy interpretation per-protein, comparison of prediction results across tools, and are ideal for downstream filtering and analysis. The Nextflow structure of metaLoc provides users with complete transparency by generating individual directories within the ‘work/’ directory for each process containing all input, intermediate, and output files. In metagenomic mode additional directories are produced in the ‘results/’ directory containing the outputs of the metagenomic mode tools used e.g. AUGUSTUS, Prodigal, and EukRep.

**Table 2:** metaLoc concise results.

Column	Workflow branch	Description
ID_merge	Both	ID of processed protein sequences
signalp_Prediction	Both	SignalP prediction of signal peptide
signalp_CS_Position	Both	SignalP prediction of signal peptide cleavage site
deeplocpro_Localization	Prokaryotic	DeepLocPro prediction of prokaryotic protein localization
deeploc_Localizations	Eukaryotic	DeepLoc prediction of eukaryotic protein localization
deeploc_Signals	Eukaryotic	DeepLoc prediction of eukaryotic protein signal peptide
deeploc_Membrane_types	Eukaryotic	DeepLoc prediction of eukaryotic protein membrane type
phobius_TM	Both (if deepTMHMM not enabled)	Phobius prediction of number of transmembrane helices
phobius_SP	Both (if deepTMHMM not enabled)	Phobius prediction of signal peptide
phobius_PREDICTION	Both (if deepTMHMM not enabled)	Phobius prediction transmembrance helices topology
deeptmhmm_Num_TM_helices	Both (if deepTMHMM enabled)	DeepTMHMM prediction of number of transmembrane helices
deeptmhmm_TM_helices(start-end)	Both (if deepTMHMM enabled)	DeepTMHMM prediction of transmembrane helices start and end positions
deeptmhmm_Prediction	Both (if deepTMHMM enabled)	DeepTMHMM prediction of protein type
deeptmhmm_Topology	Both (if deepTMHMM enabled)	DeepTMHMM prediction of protein sequence topology

All potential columns in the final concise output of metaLoc.

## 3 Conclusions

metaLoc greatly improves upon the individual protein characteristic prediction tools through the completely automatic installation and operation, and the acceptance of diverse input data. The rapidity and simplicity by which this workflow combines multiple tools makes it a valuable resource to researchers from diverse disciplines with interests in understanding the encoded protein sequences of biological datasets. With only a single command, metaLoc provides a powerful but accessible tool for researchers of varying bioinformatic experience. Furthermore, the workflow enables users to explore large datasets of proteins and the nucleotide sequences which encode them to allow streamlined bioinformatic investigations, reduced complexity of analyses, and simplified protein discovery.

## Supplementary Material

vbag169_Supplementary_Data

## Data Availability

The data and code underlying this article are available in the metaLoc GitHub repository at https://github.com/scottc-bio/metaLoc.
